# Early and Non-Invasive Detection of Chronic Wasting Disease Prions in Elk Feces by Real-Time Quaking Induced Conversion

**DOI:** 10.1371/journal.pone.0166187

**Published:** 2016-11-09

**Authors:** Yo Ching Cheng, Samia Hannaoui, Theodore R. John, Sandor Dudas, Stefanie Czub, Sabine Gilch

**Affiliations:** 1 Dept. of Ecosystem and Public Health, Faculty of Veterinary Medicine, University of Calgary, Calgary, Canada; 2 Dept. of Molecular Biology, University of Wyoming, Laramie, United States of America; 3 Canadian Food Inspection Agency, Lethbridge Laboratories, Lethbridge, Canada; Van Andel Institute, UNITED STATES

## Abstract

Chronic wasting disease (CWD) is a fatal prion disease of wild and captive cervids in North America. Prions are infectious agents composed of a misfolded version of a host-encoded protein, termed PrP^Sc^. Infected cervids excrete and secrete prions, contributing to lateral transmission. Geographical distribution is expanding and case numbers in wild cervids are increasing. Recently, the first European cases of CWD have been reported in a wild reindeer and two moose from Norway. Therefore, methods to detect the infection early in the incubation time using easily available samples are desirable to facilitate effective disease management. We have adapted the real-time quaking induced conversion (RT-QuIC) assay, a sensitive *in vitro* prion amplification method, for pre-clinical detection of prion seeding activity in elk feces. Testing fecal samples from orally inoculated elk taken at various time points post infection revealed early shedding and detectable prion seeding activity throughout the disease course. Early shedding was also found in two elk encoding a PrP genotype associated with reduced susceptibility for CWD. In summary, we suggest that detection of CWD prions in feces by RT-QuIC may become a useful tool to support CWD surveillance in wild and captive cervids. The finding of early shedding independent of the elk’s prion protein genotype raises the question whether prolonged survival is beneficial, considering accumulation of environmental prions and its contribution to CWD transmission upon extended duration of shedding.

## Introduction

Chronic wasting disease (CWD) is a prion disease which affects wild and farmed cervids such as white-tailed deer (WTD; *Odocoileus virgianus*), mule deer (MD; *Odocoileus hemionus*), elk (*Cervus canadensis*) and moose (*Alces alces*) mainly in North America and is found in 24 states of the United States as well as in 2 Canadian provinces [1; 2]. Recently, the disease was reported for the first time in Europe in a wild reindeer (*Rangifer tarandus*) and two moose in Norway [3]. Prions are infectious particles which consist only of protein, namely a beta-sheet rich isoform (PrP^Sc^) of the cellular prion protein PrP^c^ [4; 5]. Other prion diseases are Creutzfeldt-Jakob disease in humans, bovine spongiform encephalopathy (BSE) or scrapie in sheep and goats [6; 7].

Prion diseases are neurodegenerative and invariably fatal disorders which are transmissible within, but also between species, and can even be zoonotic as demonstrated for BSE which gave rise to variant CJD (vCJD) in humans [8; 9]. The risk of CWD to infect humans is considered low [10; 11], but cannot be completely excluded [12; 13]. Among cervids CWD is continuously spreading upon efficient lateral transmission [14]. The main contributing factor is that in CWD infectious prions are not confined to the central nervous system (CNS) but also found in peripheral tissues such as skeletal muscle or antler velvet [15–17]. In addition, CWD prions are shed in body fluids and excreta like saliva, urine and feces [17–19]. This results in persistent environmental contamination with prions which facilitates exposure of healthy animals to prions even without coming into direct contact with infected animals [20; 21]. Transmission among cervids is not limited by the species barrier phenomenon, however, polymorphic residues in the cervid PrP have been described which can reduce susceptibility to CWD [22]. In elk, codon 132 can encode either methionine (M) or leucine (L) [23], with methionine being the predominant allele. The presence of at least one allele encoding leucine at codon 132 (genotype 132ML or 132LL) significantly increases incubation time of CWD in both elk and transgenic mice overexpressing cervid PrP [24; 25], indicating the partially protective effect.

Given the expanding geographical distribution, annual increases in case numbers in wild cervids and the lack of effective CWD management strategies, it is of critical importance to have a test available which facilitates detection of prions at all disease stages in samples which are easily available. Such a test can then be used to support surveillance programs, which currently rely on the compliance of hunters to submit tissues of harvested animals. Brain or lymphoid tissues are used to detect PrP^Sc^ by immunohistochemistry (IHC), western blot or ELISA to recognise and confirm CWD infection [26]. Newer test developments based on the *in vitro* amplification of PrP^Sc^, such as the real-time quaking induced conversion assay (RT-QuIC; [27]) which observes amyloid formation in real time by measuring thioflavin T fluorescence over the time course of the reaction, enable the detection of CWD prions in saliva [28], urine [29], blood [30], and in feces of clinical [29] and terminally sick [31] deer. Detection by protein misfolding cyclic amplification (PMCA) of CWD prions in elk feces collected in the Rocky Mountain National Park has been reported [32]. However, a protocol to amplify CWD prions contained in fecal samples from animals that do not yet show signs of disease using RT-QuIC is still missing, but it has been demonstrated by bioassays that feces of CWD infected cervids contain infectious prions even at an asymptomatic stage of disease [18].

In the study presented here, we have refined the protocol for CWD prion detection in fecal samples by RT-QuIC, which now enables detection in feces of orally infected elk at time points post experimental infection before clinical symptoms appear. We demonstrate that fecal components inhibit RT-QuIC amplification, which can be overcome by sodium phosphotungstic acid (NaPTA) precipitation [33] of PrP^Sc^ prior to RT-QuIC. Using recombinant mouse PrP as a substrate improves the detection of CWD prions. In combination with substrate replacement, sufficient sensitivity of RT-QuIC and reduction of spontaneous conversion of recombinant PrP (rPrP) substrate was achieved to specifically amplify prion seeds in fecal samples of orally infected elk. We were able to detect seeding activity at early time points after inoculation, and shedding was detected in samples obtained from individual animals throughout the incubation time and clinical stage of the disease. Encoding a leucine at amino acid position 132 which is linked to partial genetic resistance to CWD infection [24; 25] did not prevent early shedding.

In summary, our results demonstrate that testing of cervid fecal samples by RT-QuIC can be a versatile tool to support CWD surveillance. The finding that shedding can start early after oral infection even in elk which are heterozygous at codon 132 (132LM) indicates that environmental contamination with CWD prions upon fecal shedding might be even more significant than anticipated previously, and questions the benefit of alleles that reduce susceptibility to CWD despite prolonged survival time.

## Materials and Methods

### Animals and Fecal Sampling

All animal work strictly followed the guidelines of the Canadian Council for Animal Care (CCAC) and was approved by the Canadian Food Inspection Agency (CFIA) and the Lethbridge Laboratory Animal Care Committee (Protocol # 04002). Samples used in this study were obtained from a previous elk CWD pathogenesis study. All animals were housed in the BSL3 large animal indoor containment facility at the CFIA National Centre for Animal Disease Lethbridge. After an adjustment period to the new environment for one week, elk were challenged orally with 1g of confirmed CWD positive (18 animals) or negative (5 animals) elk brain tissue. At defined time points post inoculation animals were euthanized by injection of an overdose of pentobarbital. CWD status was confirmed post mortem by immunohistochemistry of intestinal lymph nodes and immunoblot [34; 35]. Fecal samples were taken from the rectum during necropsy in order to avoid cross-contamination of samples in the holding pens.

### Preparation of Brain Homogenates

Brain homogenates were prepared to a final concentration of 10% (w/v) in phosphate-buffered saline pH 7,4 (PBS) using a dounce homogenizer. Aliquots were stored at -80°C. Brains of experimentally infected C57Bl/6 mice (strain 22L), elk or mule deer were used.

### Preparation of Fecal Extracts

Fecal pellets were weighed and prepared in fecal extract buffer (20 mM sodium phosphate (pH 7.1), 130 mM NaCl, 0.05% Tween 20, 1mM PMSF and 1X complete protease inhibitors (Roche) at a final concentration of 10 or 20% (w/v). Fecal pellets were homogenized (GentleMACS dissociator, Miltenyi Biotec) and placed onto a rotary shaker for 1 hour at room temperature. After centrifugation at 18,000 x g for 5 mins, supernatants were collected for further use.

### Spiking Experiments

Fecal samples were obtained from a confirmed CWD-negative mule deer population in Pullman, WA, USA. Homogenates were prepared in feces extract buffer at a concentration of 10 or 20% (w/v). CWD positive mule deer brain homogenate was added to a final concentration of 1% and 10x serial dilutions were prepared.

### Sodium Phosphotungstic Acid (NaPTA) Precipitation

One ml of fecal homogenate was mixed with N-lauryl-sarcosine at a final concentration of 2% and incubated at 37° C and constant shaking at 1,400 rpm for 30 min. The samples were adjusted to 0.3% NaPTA by adding a stock solution containing 4% NaPTA and 170 mM MgCl2, pH7.4, incubated for 2 hours at 37° C with constant shaking and centrifuged for 30 min at maximum speed (15,800 x g) at room temperature. Pellets were washed using cell lysis buffer containing 0.1% N-lauryl-sarcosine, and then resuspended in 1/10 of the original sample volume in RT-QuIC dilution buffer.

### Expression and Purification of Recombinant Prion Proteins

Mature forms of deer (aa 24–234; construct kindly provided by Dr. B. Caughey, NIH Rocky Mountain Laboratories, Hamilton, MT) or mouse (aa 23–231) PrP were cloned into pET expression vectors and expressed in *E*. *coli* Rosetta using the Express Autoinduction System (Novagen). Inclusion bodies were prepared using the Bug Buster reagent (Novagen) and solubilized in lysis buffer (guanidine-HCl 8 M, Na-phosphate 100 mM, Tris-HCl 10 mM, pH 8.0) for 50 min at room temperature and then centrifuged at 16,000 x g for 5 min at room temperature. Binding, refolding and elution using an AKTA Explorer system has been described [29].

### RT-QuIC Assay and Substrate Replacement

Real time QuIC was performed as described [27; 29]. Briefly, reactions were set up in assay buffer containing 20 mM sodium phosphate (pH 6.9), 300 mM NaCl, 1 mM EDTA, 10 μM Thioflavin T and 0.1 mg/ml rPrP substrate. Ninety-eight μl aliquots were added to the wells of a 96 well optical bottom plate (Nalge Nunc International). Quadruplicate reactions were seeded with 2 μl of brain homogenate or feces extract that were diluted in 20 mM sodium phosphate (pH 6.9), 130 mM NaCl, 0.1% (w/v) sodium dodecyl sulfate (SDS), 1X N2 Supplement (Invitrogen). The plate was sealed with Nunc Amplification Tape (Nalge Nunc International) and placed in a BMG Labtech FLUOstar Omega fluorescence plate reader that was pre-heated to 42° C for a total of 50 hours of cycling. Fluorescence values were plotted as the average of quadruplicate reactions (GraphPad Prism software). For substrate replacement, cycling was stopped after 25 h and 90 μl of the reaction mixture were replaced with fresh assay buffer containing rPrP and cycling was continued for further 50 hours. Samples were scored positive if at least 50% of the replicates [36] reached a ThT fluorescence cut-off, which was calculated based on the average ThT fluorescence plus 5x standard deviations [37].

## Results

Current diagnosis and surveillance of CWD in wild cervids are mostly relying on hunters’ compliance to submit heads of harvested animals to have brain homogenates tested for PrP^Sc^. Pre-mortem detection of PrP^Sc^ is mainly restricted to using biopsies of lymphoid tissues, which requires the stressful procedure of capturing wild cervids to obtain samples. Therefore, we have decided to employ fecal samples as seeding material in RT-QuIC to test for CWD.

We and others have recently provided proof-of-principle that detection of CWD seeding activity in cervid fecal samples by RT-QuIC is possible [29; 31]. Sensitivity of detection was low, which is evident from a long lag phase of the reaction and low ThT fluorescence values [29] and only demonstrated for samples from animals at late stages of the disease [29; 31]. However, a test to detect CWD infection in fecal samples before clinical symptoms appear would be desirable.

To verify whether fecal contents may contain inhibitors of amyloid formation we performed spiking experiments. Fecal samples from a confirmed CWD-free population were homogenized in feces extract buffer to a final concentration of 10 or 20% and spiked with CWD-positive brain homogenate. Serial dilutions were prepared and used to seed RT-QuIC assay with deer rPrP as a substrate. CWD-positive brain served as a control. Whereas in 20% fecal homogenates all reactions were negative, in 10% fecal homogenates the seeding activity was detectable up to a dilution of 2 x 10^−6^, similar to the detection limit in the CWD-positive brain homogenate (S1A Fig). Whereas in CWD-negative brain homogenate no signal was detectable (S1A Fig), in undiluted or 2x10^-1^ diluted 10% fecal homogenates of non-infected elk an increase in ThT-fluorescence after 30 hours, likely due to spontaneous conversion of deer rPrP, was observed (S1B Fig). This indicates that 10% fecal homogenate as a substrate favours spontaneous conversion more compared to CWD-negative brain homogenate (S1A Fig).

**Fig 1:**
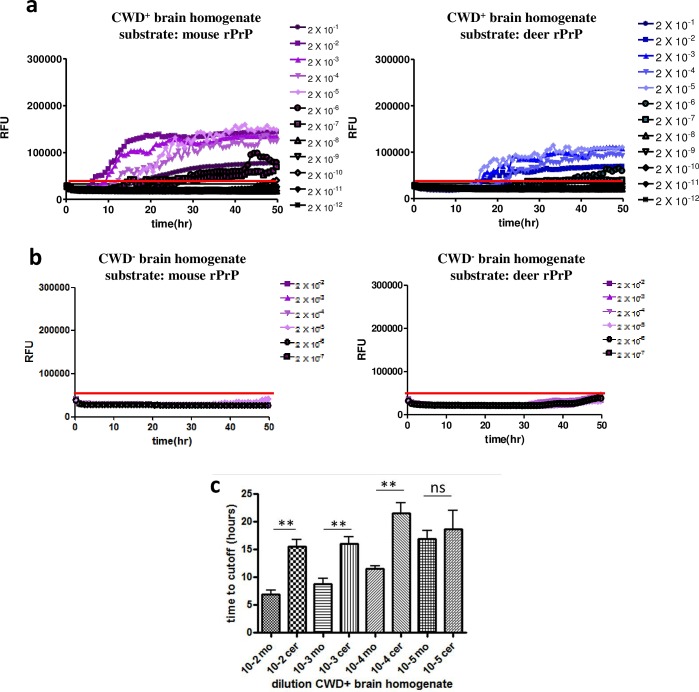
Mouse rPrP is a more efficient substrate for amplification of CWD seeding activity. Serial dilutions of CWD-positive **(a)** or negative **(b)** elk brain homogenates were used as seeds for RT-QuIC reactions. As a substrate, either mouse (left panel) or deer rPrP (right panel) were used. Y-axes indicate the relative ThT fluorescence units, x-axes show the reaction time. A threshold to determine positive reactions was calculated by using the average baseline fluorescence plus 5 standard deviations, which is indicated as a solid line at app. 50,000 RFU. **(c)** The time to reach the threshold (y-axis) was determined for each individual reaction. Dilutions of 2x10^-2^ to 2x10^-5^ of reactions with either mouse or deer rPrP were included, and the averages of the time to reach the threshold are shown. Bars represent standard deviations. For each dilution mouse and deer rPrP were compared and the difference between the two groups was statistically evaluated using unpaired student’s t-test (GraphPad Prism software; ** = p-value < 0.01; ns = not significant).

Therefore, we tested a different rPrP substrate in order to reduce the rate of spontaneous conversion which can result in false-positive scoring of samples. We chose mouse rPrP (aa23-231) which has been used previously to detect CWD prions [38]. First, we compared the efficiency of mouse and deer rPrPs as a substrate to amplify CWD prions. CWD-infected elk brain homogenate was serially diluted and used to seed RT-QuIC reactions with either mouse or deer rPrP as a substrate. Whereas with mouse rPrP seeding activity was detectable up to a dilution of 2x10^-6^, the endpoint with deer rPrP was reached at 2x10^-5^ (Fig 1A), taking into account only dilutions for which at least 50% of the replicates exceeded the threshold fluorescence level (average of baseline ThT fluorescence + 5 standard deviations (SD); [37]). Reactions seeded with CWD-negative brain homogenates served as a control. Here, a slight increase of ThT fluorescence was observed when deer rPrP was used as a substrate, which was less pronounced with mouse rPrP (Fig 1B). In RT-QuIC reactions seeded with CWD-positive brain homogenate dilutions, the reaction time necessary to reach the threshold (lag phase), was significantly shorter (about 10 hours; p-value < 0.01) with mouse rPrP substrate for dilutions between 2x10^-2^ to 2x10^-4^, (Fig 1C). A shorter lag phase (p-value > 0.05) with mouse rPrP was also observed for the 2x10^-5^ dilution. This was confirmed when we used WTD CWD isolates to seed RT-QuIC reactions (data not shown). Althogether, these data indicate that mouse rPrP as a substrate facilitates an improved CWD prion amplification compared to deer rPrP.

**Fig 2:**
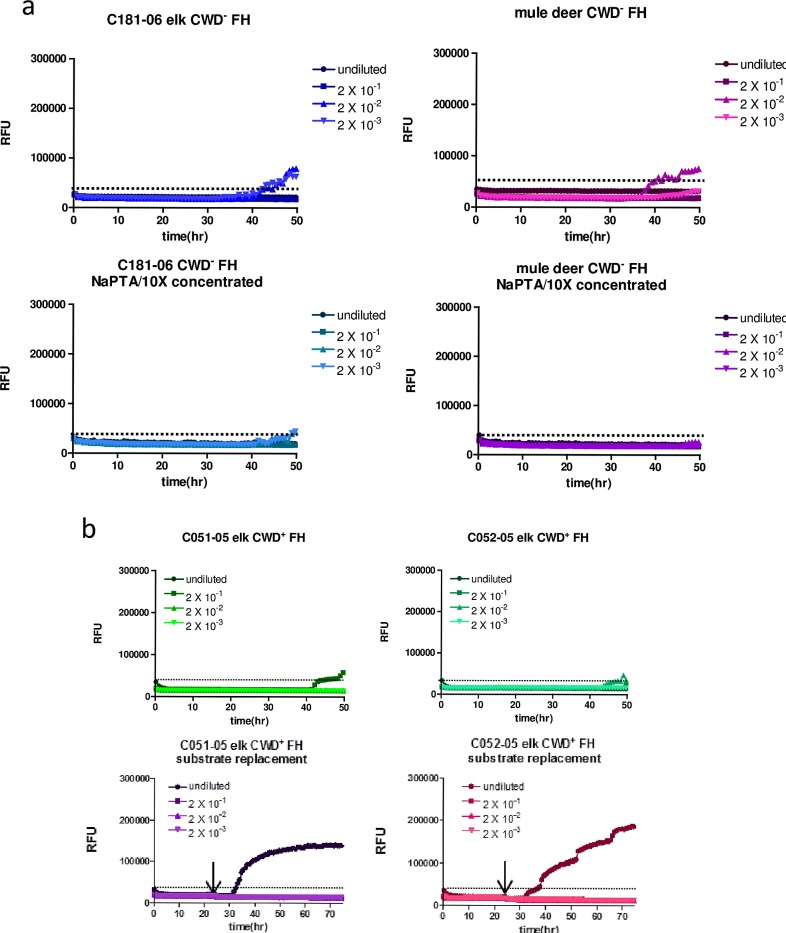
NaPTA precipitation and substrate replacement enable detection of CWD seeding activity in feces. **(a)** Fecal homogenates (10% w/v) of non-infected elk or mule deer were subjected to NaPTA precipitation and 10fold concentration (lower panel) or not (upper panel). These samples were either used undiluted or in dilutions as indicated for seeding RT-QuIC reactions with mouse rPrP as a substrate. The y-axes show relative ThT fluorescence units, the x-axes depict the reaction time. **(b)** Fecal homogenates of two individual elk which were orally infected with CWD were subjected to NaPTA precipitation and 10fold concentration. RT-QuIC reactions with mouse rPrP as a substrate were run for 50 hours (upper panel), or for 75 hours (lower panel). For the latter, the reaction was stopped after 25 hours, and 90% of the reaction volume was removed and replaced by freshly prepared RT-QuIC mix containing rPrP substrate and ThT. Then the RT-QuIC assay was continued.

In order to remove fecal contents, to concentrate PrP^Sc^ seeds and to further reduce spontaneous conversion which occurred also with mouse rPrP in RT-QuIC reactions using fecal homogenates of CWD-negative elk or deer (Fig 2A; upper panels), we introduced NaPTA precipitation of fecal homogenates. This did not interfere with detection of seeding activity in prion-infected brain homogenates from various species but appeared to improve detection, with overall higher ThT fluorescence values (S2A Fig). NaPTA precipitates were re-suspended in 1/10 of the original sample volume resulting in a 10x concentration of seeding activity. Using these conditions, spontaneous conversion of mouse rPrP was strongly reduced and only observed at very low levels and late time points after > 45 hours reaction time, with ThT fluorescence values that did not reach the threshold value and are therefore considered negative (Fig 2A; lower panels). Notably, in reactions with deer rPrP as a substrate spontaneous conversion was evident even after NaPTA-precipitation and concentration of CWD-negative fecal homogenates (S2B Fig).

As only a weak increase of ThT fluorescence was found in fecal homogenates of CWD-infected elk (Fig 2B, upper panel), further improvement of sensitivity was achieved by substrate replacement after 25 hours reaction time (Fig 2B, lower panel). Fecal samples collected at the clinical stage of disease from elk orally infected with CWD in an experimental pathogenesis study [34; 35] were used as a seed either undiluted or serially diluted up to 2x10^-3^. With substrate replacement after 25 hours and continued reaction for 50 hours, the lag phase was significantly shortened from more than 40 hours to less than 35 hours. After addition of fresh substrate, the amplification of amyloid was highly efficient; this was evident by the steep increase of ThT fluorescence (Fig 2B).

**Fig 3:**
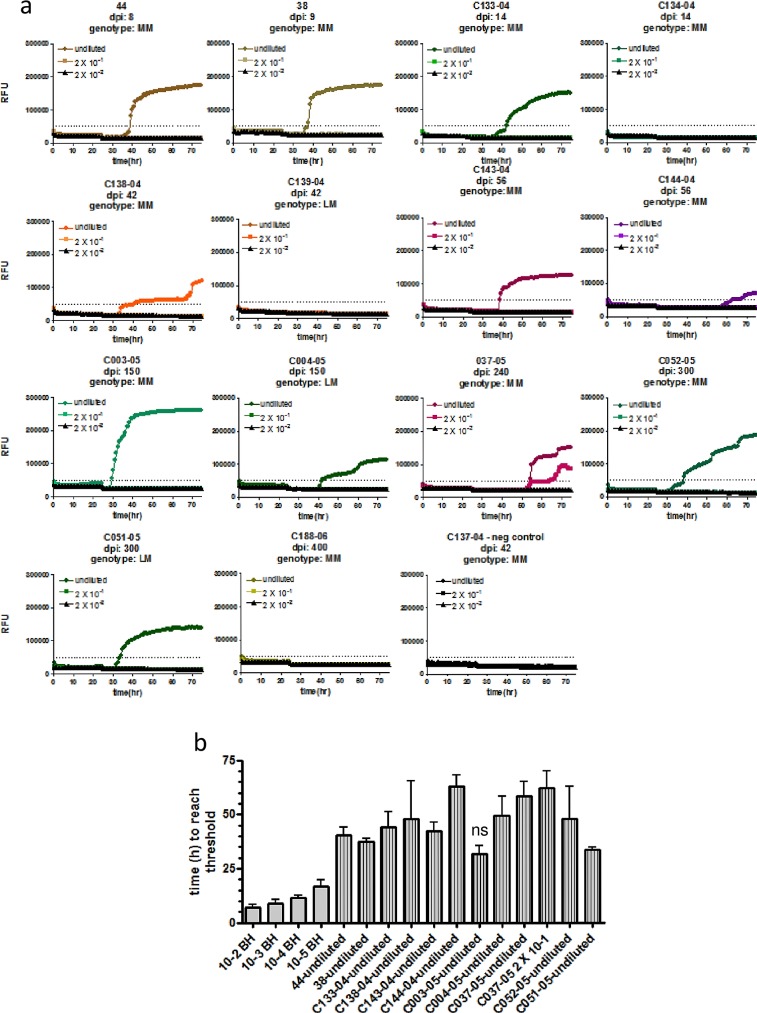
CWD seeding activity is detectable in fecal samples of elk at the pre-clinical stage of disease. **(a)** Fecal homogenates (10% w/v) of elk orally infected with CWD prions and taken after different time points post infection (dpi) were subjected to NaPTA precipitation and 10fold concentration. These samples were either used undiluted or in two serial dilutions as indicated to seed RT-QuIC reactions with mouse rPrP as a substrate. Substrate replacement was done after a reaction time of 25 hours. The *Prnp* genotype is indicated, with MM animals being homozygous for methionine at amino acid 132, LM indicates heterozygosity for methionine/leucine. **(b)** The time to reach the threshold (lag time) was determined for each positive sample and dilutions of 2x10^-2^ to 2x10^-5^ of the CWD-positive elk brain homogenate. Averages of 4 replicates are shown, bars represent the standard deviation. Statistical analysis was done using ANOVA and post-hoc analysis by Dunnett’s Multiple Comparison Test (ns: p-value > 0.05; GraphPad Prism software).

**Fig 4:**
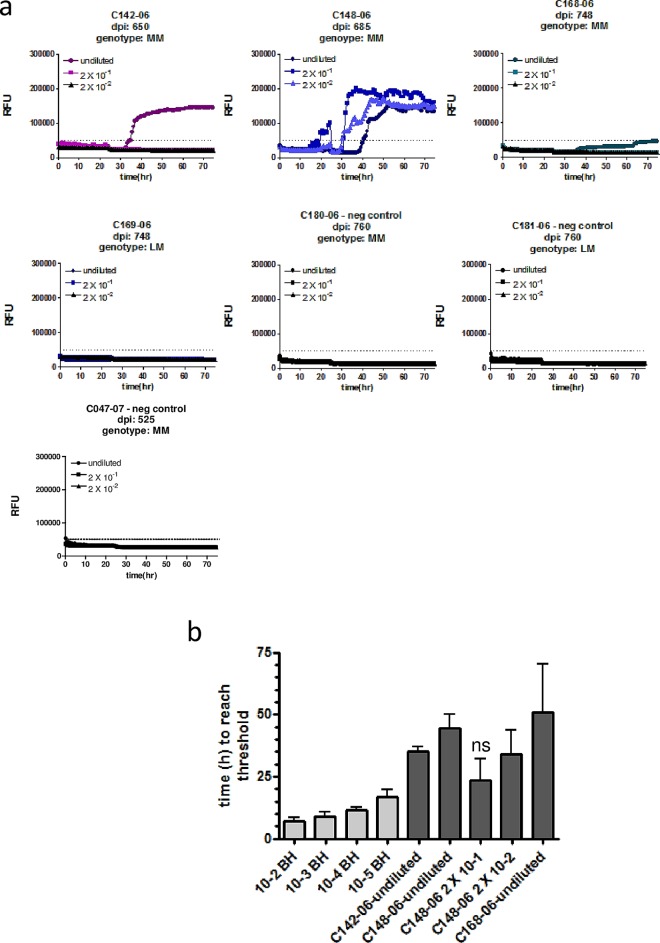
CWD seeding activity is detectable in fecal samples of elk at the clinical stage of disease. **(a)** Fecal homogenates (10% w/v) of elk orally infected with CWD prions and taken after different time points post infection (dpi) were subjected to NaPTA precipitation and 10fold concentration. These samples were either used undiluted or in two serial dilutions as indicated to seed RT-QuIC reactions with mouse rPrP as a substrate. Substrate replacement was done after a reaction time of 25 hours. The *Prnp* genotype is indicated, with MM animals being homozygous for methionine at amino acid 132, LM indicates heterozygosity for methionine/leucine. **(b)** The time to reach the threshold (lag time) was determined for each positive sample and dilutions of 2x10^-2^ to 2x10^-5^ of the CWD-positive elk brain homogenate. Averages of 4 replicates are shown, bars represent the standard deviation. Statistical analysis was done using ANOVA and post-hoc analysis by Dunnett’s Multiple Comparison Test (ns: p-value > 0.05; GraphPad Prism software).

Our ultimate goal was to determine whether shedding of CWD prions in fecal samples can be detected by RT-QuIC throughout the time course of infection. We employed fecal samples collected from individual animals at different time points after experimental oral inoculation of elk with CWD-infected brain homogenate. The genotype at polymorphic codon 132 (M/L) has been determined [34; 35], and elk carrying 132MM and 132LM alleles were included in the study. We have tested samples from 18 elk, 14 samples from animals at the pre-clinical stage of disease and 4 samples from elk with clinical CWD. Samples from four mock-inoculated elk and two deer from a confirmed CWD-negative population were used as negative controls (Figs 3 and 4 and S3 Fig), and were included in each RT-QuIC assay.

All samples were used in RT-QuIC either undiluted, or diluted 2x10^-1^ and 2x10^-2^. Samples were scored positive if at least 50% of the replicates exceeded the threshold ThT fluorescence. Of the 14 preclinical samples obtained between 8 and 400 days post infection (dpi; Fig 3), 11 samples (78.5%) were positive in RT-QuIC, including at least one out of two samples taken at very early time points (8, 9 or 14 dpi). Only one out of eleven samples (C037-05; 240 dpi; 132MM) was positive in the 1:20 dilution, in all other samples seeding activity was detected only in the undiluted sample (Fig 3A). The duration of the lag phase (i.e. time to reach the threshold) was between 30 and 60 hours, and for all samples except C003-05 (150 dpi; 132MM) significantly longer (p-value < 0.01) than the lag phase of the 2x10^-5^ dilution of CWD-positive brain homogenate (Fig 3B), indicating that significantly lower amounts of seeding activity are contained in fecal samples. Three samples were from elk with a heterozygous genotype (132LM). Of these, the sample taken at 42 dpi was negative, whereas in samples collected at 150 and 300 dpi, respectively, prion seeding activity was detectable. The average time to reach the threshold was similar between codon 132 heterozygous and homozygous elk (Fig 3B). All animals used in this study were tested by IHC using brain and intestinal lymph node samples, and the earliest accumulation of PrP^Sc^ in intestinal lymph nodes was found in elk C052-05 (132MM) at 300 dpi. All animals euthanized before 300 dpi were negative, elk euthanized after 300 dpi were positive [34; 35].

From the undiluted samples of animals with clinical signs of disease, three out of four samples (75%) were positive (Fig 4A). Although the average ThT fluorescence in sample C168-06 only slightly exceeds the threshold, in 50% of the replicates ThT fluorescence above the cut-off was measured and therefore this sample was scored positive. All positive samples were from elk homozygous for methionine. Sample C148-06 (685 dpi; 132MM) was positive in all tested dilutions up to 2x10^-2^, and the average duration of the lag phase of the 2x10^-1^ dilution was not significantly different to that of the 2x10^-5^ dilution of the CWD-positive elk brain homogenate (Fig 4B). Interestingly, this animal died at 685 dpi because of a significant wasting syndrome and severe ataxia (S. Gilch and S. Czub, personal communication). In contrast to the 132MM homozygous animals, elk C169-06 (132LM) exhibited signs of only early clinical disease at 748 dpi demonstrating the protective effect of the leucine allele, and no seeding activity was detectable (Fig 4A).

RT-QuIC results shown in Fig 3 and Fig 4, the elk’s *Prnp* genotypes and the time points of necropsy and sample collection are summarized in Table 1.

**Table 1:** Summary of RT-QuIC results.

Animal ID	Genotype codon 132	dpi	RT-QuIC result/endpoint
N/A	MM	8	pos/undiluted
N/A	MM	9	pos/undiluted
C133-04	MM	14	pos/undiluted
C134-04	MM	14	neg
C138-04	MM	42	pos/undiluted
C139-04	LM	42	neg
C143-04	MM	56	pos/undiluted
C144-04	MM	56	pos/undiluted
C003-05	MM	150	pos/undiluted
C004-05	LM	150	pos/undiluted
C037-05	MM	240	pos/2 x 10^−1^
C051-05	LM	300	pos/undiluted
C052-05	MM	300	pos/undiluted
C188-06	MM	400	neg
C142-06*	MM	650	pos/undiluted
C148-06*	MM	685	pos/2 x 10^−2^
C168-06*	MM	748	pos/undiluted
C169-06**	LM	748	neg

* clinical disease

** early clinical disease

**Fig 5:**
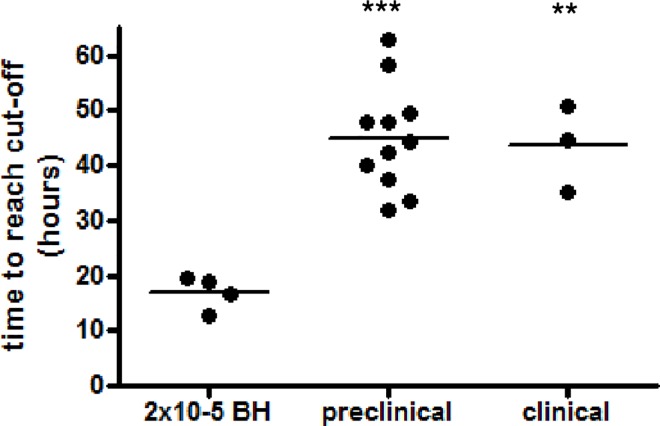
Significantly prolonged lag times in RT-QuIC positive feces from pre-clinical and clinical elk. The average lag time of undiluted RT-QuIC positive samples from individual animals was compared to the lag time of a 2x10^-5^ dilution of CWD-positive brain homogenate. Statistical analysis was done using ANOVA and post-hoc analysis by Bonferroni Multiple Comparison Test (ns: p-value > 0.05; **: p-value <0.05; GraphPad Prism software).

On average, the duration of the lag phases of undiluted positive samples did not significantly differ between samples from elk at the pre-clinical and clinical stages of CWD. However, the average time to reach the threshold of both pre-clinical and clinical samples was significantly longer (p-value < 0.01) than for the 2x10^-5^ dilution of the CWD-positive elk brain homogenate (Fig 5).

In summary, we have established a RT-QuIC protocol for detection of CWD seeding activity in fecal samples obtained at the pre-clinical stage of the disease. We demonstrate that elk after experimental oral challenge with CWD-infected brain homogenate shed detectable amounts of seeding activity in feces as early as 14 dpi and throughout the incubation time and clinical stage of the disease. Shedding was independent of the elk’s genotypes at codon 132.

## Discussion

Options for pre-mortem diagnosis of CWD infection using specimen that are easily available are limited. We have designed this study to adapt RT-QuIC, a highly sensitive assay with high-throughput capacity for detection of CWD prion seeds in fecal samples. This test can be useful in the future to support CWD surveillance programs by analysing fecal samples collected in the field. This will require to evaluate the influence of environmental factors such as drying, aging, rain fall on the detection of CWD prions in feces. Another application can be the testing of farmed cervids. We have employed fecal samples of elk orally challenged with CWD prions to determine shedding at different time points after oral infection. Our results show that prion seeding activity is detectable in samples collected at the asymptomatic or clinical stages of the disease.

When adapting the RT-QuIC assay for the use with cervid fecal samples we found that amyloid formation was inhibited by fecal components. Most likely bile acids and/or derivatives are among the components mainly responsible for this. Secondary and taurine-conjugated forms of bile acids UDCA (ursodeoxycholic acid) and TUDCA (tauroursodeoxycholic acid) exhibit anti-aggregation effects on guanidinium-induced *in vitro* fibrillization of rPrP and in RT-QuIC assay when added to the reaction [39]. This was evident for three different prion strains by a significantly prolonged lag phase and a reduced ThT fluorescence [39]. To avoid this effect, we employed NaPTA precipitation [33] which enables purification and concentration of prion seeds and which has been used previously in RT-QuIC for detection of seeding activity in blood, urine or saliva [28;30;40]. NaPTA precipitation with an incubation period of 2 hours did not interfere with RT-QuIC using brain homogenates and even led to an increase of the maximum ThT fluorescence.

The concept of species barrier suggests that the identity of the PrP primary structure is a main factor for predicting transmissibility of prion disease between species [41; 42]. We show here that mouse rPrP is better suited for amplification of CWD prions than cervid PrP, indicating that amplification in RT-QuIC does not follow the rules of the species barrier concept, as observed previously [43; 44]. We also found a higher spontaneous conversion rate for cervid rPrP, possibly because of the ‘rigid loop’ structure [45; 46] which makes PrP more aggregation-prone [38]. In a previous study, spontaneous conversion of mouse rPrP was reported when the reaction was performed under conditions we used with 300 mM NaCl in the reaction buffer, and with a 5x10^-6^ dilution of non-infected brain homogenate as a seed [47]. We filtered the rPrP substrate before and after dialysis [29] rather than only before dialysis as in the protocol cited by Vascellari et al. [27], which might help to prevent spontaneous conversion of mouse rPrP under our experimental conditions. To further improve the detection of CWD seeding activity, a substrate replacement step after a reaction time of 25 hours was introduced as described for enhanced RT-QuIC (eQuIC; [48]). Using mouse rPrP substrate (aa 90–231) this step did not increase sensitivity of detection of different mouse scrapie strains [47]. Our results demonstrating the successful use of substrate replacement with mouse rPrP (aa 23–231) for fecal samples indicate that the beneficial effect may also depend on the source of the prions used for seeding in addition to the previously discussed influence of the rPrP substrate [47].

The presence of prions in fecal samples of cervids [18; 32] and sheep [49] has been demonstrated by mouse bioassays or PMCA. In rodents fecal prions were detected by various methods mainly hours up to days after oral inoculation, indicating gut-passaged prions that were not absorbed [50; 51]. In feces of elk from a CWD endemic area in the Rocky Mountain National Park, PMCA-positive samples were found that originated from animals which also showed positive for IHC staining in lymphoid tissue, whereas only one sample was positive without confirmatory IHC [32].

In our study, we have employed samples collected at defined time points during an experimental oral CWD transmission study involving elk encoding PrP homozygous for MM or heterozygous for LM at codon 132. Animals were challenged with a dose that was similar to a dose that caused a 100% attack rate upon oral inoculation of red deer (*Cervus elaphus*) with CWD from elk despite an amino acid difference between elk and red deer at codon 226 [52]. Therefore we can assume that all elk used in our study were successfully infected and eventually would have developed disease. Our results show consistent shedding throughout the incubation period, independent of the *Prnp* genotype. Several samples (three obtained at the preclinical and one collected at the clinical stage) were negative in RT-QuIC. This can indicate that either these samples did not contain seeding activity or that the assay was not sensitive enough to detect the seeding activity. However, options to confirm that the samples do not contain PrP^Sc^ are limited. Even in indicator mouse bioassays which were used to demonstrate the presence of CWD prions in feces of asymptomatic deer, samples from one animal obtained at the clinical stage did not cause diseases in mice upon intracerebral infection, whereas samples from the same animal collected at the asymptomatic stage caused an up to 50% attack rate in mice [18]. In contrast, the specificity of detection was high, as we were able to efficiently circumvent spontaneous conversion by introducing mouse rPrP substrate, NaPTA precipitation and substrate replacement. Negative controls were included in every experiment, and none of our negative controls exhibited spontaneous conversion at a level to be considered positive by our criteria, which encompass that 50% of the replicates have to exceed the threshold fluorescence. Therefore, although we currently cannot exclude that the RT-QuIC negative samples are false negative, we conclude that the false-positive rate is extremely low.

Shedding in feces at the pre-clinical stage has been demonstrated earlier by bioassays using feces of mule deer orally infected with CWD as an inoculum for intracerebral inoculation of mice [18]; in this study infectivity was found in fecal samples obtained 9 months post infection, and the amount of prion infectivity detected in feces did not increase over time from the pre-clinical to the clinical disease stage [18]. In accordance with this, and although longitudinal data are missing in our study, we have not observed a significant reduction in the time to reach the threshold which can serve as an indicator for the amount of prions, in samples obtained from individual animals that were euthanized later after inoculation compared to samples taken early post infection. We detected seeding activities already at 14 and 21 dpi in one out of the two samples taken at each time point, long before PrP^Sc^ detection by IHC in lymphoid tissue samples. Positive results at 8 and 9 dpi were most likely due to residual inoculums. Considering that passage of gut contents in cervids is completed within 14 days [53] and that one sample at each time point (14 and 42 dpi) was negative supports the assumption that these and all later samples are not the result of residual inoculum. Abnormal PrP was detectable in follicular germinal centres of the retropharyngeal lymph nodes, Peyer’s patches and ileocaecal nodes of mule deer as early as 42 days post oral inoculation when using an improved IHC staining protocol, although the percentage of positive follicles was very low (between 0.53% and 6.7%) [54]. In mouse bioassays replication of PrP^Sc^ in intestinal follicular dendritic cells was directly detected by an increased PrP^Sc^ immunostaining as early as 7–21 days post oral inoculation [55]. Therefore, it appears likely that a reservoir of PrP^Sc^ replication exists in peripheral organs, possibly in lymphoid tissue, from which seeding activity is constantly released and transported into the gut lumen.

Unfortunately, only a very limited number of animals with a 132LM genotype were included in this study which allows only drawing preliminary conclusions from those samples. We found two out of three samples taken at the pre-clinical stage (150 or 300 dpi) to be positive in RT-QuIC. Duration of the RT-QuIC lag phase of samples from heterozygous elk was similar to that of homozygous animals, indicating similar amounts of seeding activity, although the efficiency of rPrP conversion might different for prion seeds originating from an animal expressing a non-wildtype allele of PrP. Only one sample from a heterozygous elk euthanized with early clinical signs at 748 dpi was available which was negative but the observation of disease symptoms demonstrates that eventually the heterozygous elk in this study developed clinical disease.

This is the first study which addresses fecal shedding in the context of the PrP genotype at codon 132 in elk and despite the limited number of samples, the positive results for those obtained from pre-clinical animals demonstrates that heterozygous elk shed prions in feces early after infection and before clinical signs become evident. For shedding of CWD prions in urine and saliva of white-tailed deer carrying either the 96GG or the less frequent 96GS genotypes [40], no differences in frequency and amounts of seeding activity shed by the animals during pre-clinical and clinical stages were observed [40]. The incubation time of CWD in elk carrying the 132LM alleles is almost twice as long as for animals which are homozygous for methionine [24].

Altogether, we provide new insights into the fecal shedding of CWD prions in elk. The early release of prions in feces suggests ongoing replication in a prion reservoir in peripheral tissues. Our results raise the question how beneficial heterozygosity for alleles which increase incubation times is despite extended survival times, taking into account the prolonged time of environmental contamination if shedding starts early at an asymptomatic stage.

## Supporting Information

S1 Fig Fecal homogenate inhibits detection of CWD prions by RT-QuIC. **(a)** Twenty or 10% (w/v) fecal homogenate was prepared in feces extract buffer using samples collected from a CWD-negative elk. The homogenates were spiked with brain homogenate from a CWD-infected elk at a final concentration of 1%. Aliquots of 10fold serial dilutions thereof as indicated were analysed by RT-QuIC using deer PrP as a substrate. Serial dilutions of CWD-positive or negative brain homogenate served as controls. **(b)** Serial dilutions of fecal homogenates (10%) of CWD-negative elk were used to seed RT-QuIC reactions with deer rPrP as a substrate. All assays were performed in quadruplicate, x-axes show the reaction time, y-axes the relative thioflavin T (ThT) fluorescence.
(PDF)

S2 Fig NaPTA precipitation does not inhibit RT-QuIC detection of prion seeds in infected brain homogenates. **(a)** Prion-infected brain homogenates of white-tailed deer, mule deer and mouse were subjected to NaPTA precipitation (lower panel) or not (upper panel). NaPTA precipitates were re-suspended in the original volumes. NaPTA-precipitated samples and non-precipitated brain homogenates were serially diluted, and aliquots of dilutions were used to seed RT-QuIC reactions in quadruplicate. Deer rPrP was used as a substrate. **(b)** Ten % fecal homogenates of CWD-negative elk were subjected to NaPTA precipitation and 10fold concentration. Serial dilutions were tested by RT-QuIC with deer rPrP as a substrate. Reactions were set up in quadruplicate, average ThT fluorescence is shown over the time course of the reaction.
(PDF)

S3 Fig RT-QuIC with fecal homogenates of CWD-negative deer. As further negative controls, fecal homogenates of CWD-negative deer were subjected to NaPTA precipitation and 10fold concentration. Serial dilutions were used to seed RT-QuIC reactions with mouse rPrP and substrate replacement. Reactions were set up in quadruplicate, average ThT fluorescence is shown over the time course of the reaction.
(PDF)
