# Supplementary material for: Early and Non-Invasive Detection of Chronic Wasting Disease Prions in Elk Feces by Real-Time Quaking Induced Conversion
Source: PLoS One. 2016 Nov 9;11(11):e0166187. doi: 10.1371/journal.pone.0166187 (PMC5102397; doi:10.1371/journal.pone.0166187)
Supplement: S1 Fig — (a) Twenty or 10% (w/v) fecal homogenate was prepared in feces extract buffer using samples collected from a CWD-negative elk. The homogenates were spiked with brain homogenate from a CWD-infected elk at a final concentration of 1%. Aliquots of 10fold serial dilutions thereof as indicated were analysed by RT-QuIC using deer PrP as a substrate. Serial dilutions of CWD-positive or negative brain homogenate served as controls. (b) Serial dilutions of fecal homogenates (10%) of CWD-negative elk were used to seed RT-QuIC reactions with deer rPrP as a substrate. All assays were performed in quadruplicate, x-axes show the reaction time, y-axes the relative thioflavin T (ThT) fluorescence. (PDF) [file pone.0166187.s001.pdf]

S1 Figure

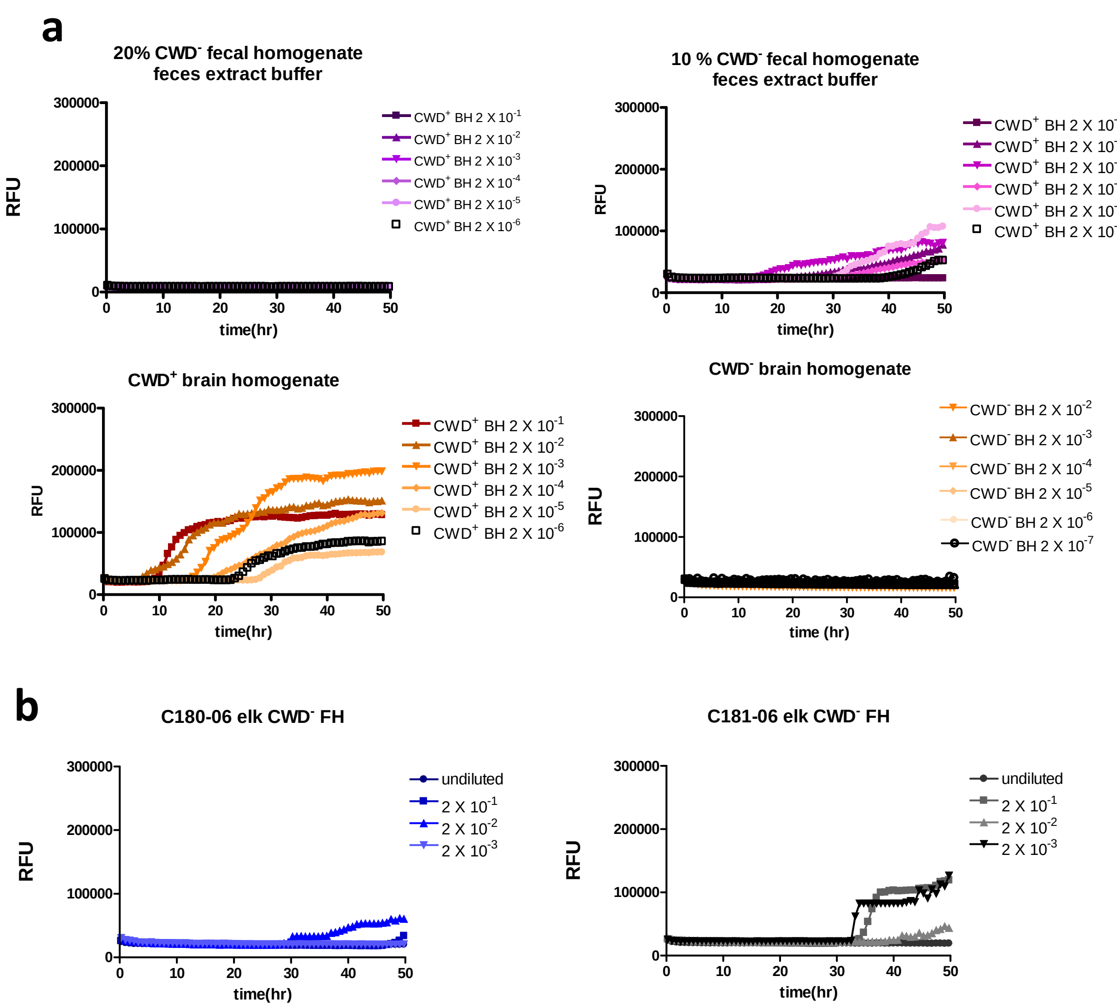

Fecal homogenate inhibits detection of CWD prions by RT-QuIC. (a) Twenty or 10 % (w/v) fecal homogenate was prepared in feces extract buffer using samples collected from a CWD-negative elk. The homogenates were spiked with brain homogenate from a CWD-infected elk at a final concentration of 1 %. Aliquots of 10fold serial dilutions thereof as indicated were analysed by RT-QuIC using deer PrP as a substrate. Serial dilutions of CWD-positive or negative brain homogenate served as controls. **(b)** Serial dilutions of fecal homogenates (10 %) of CWD-negative elk were used to seed RT-QuIC reactions with deer rPrP as a substrate. All assays were performed in quadruplicate, x-axes show the reaction time, y-axes the relative thioflavin T (ThT) fluorescence.
