# Supplementary material for: Early and Non-Invasive Detection of Chronic Wasting Disease Prions in Elk Feces by Real-Time Quaking Induced Conversion
Source: PLoS One. 2016 Nov 9;11(11):e0166187. doi: 10.1371/journal.pone.0166187 (PMC5102397; doi:10.1371/journal.pone.0166187)
Supplement: S2 Fig — (a) Prion-infected brain homogenates of white-tailed deer, mule deer and mouse were subjected to NaPTA precipitation (lower panel) or not (upper panel). NaPTA precipitates were re-suspended in the original volumes. NaPTA-precipitated samples and non-precipitated brain homogenates were serially diluted, and aliquots of dilutions were used to seed RT-QuIC reactions in quadruplicate. Deer rPrP was used as a substrate. (b) Ten % fecal homogenates of CWD-negative elk were subjected to NaPTA precipitation and 10fold concentration. Serial dilutions were tested by RT-QuIC with deer rPrP as a substrate. Reactions were set up in quadruplicate, average ThT fluorescence is shown over the time course of the reaction. (PDF) [file pone.0166187.s002.pdf]

S2 Figure

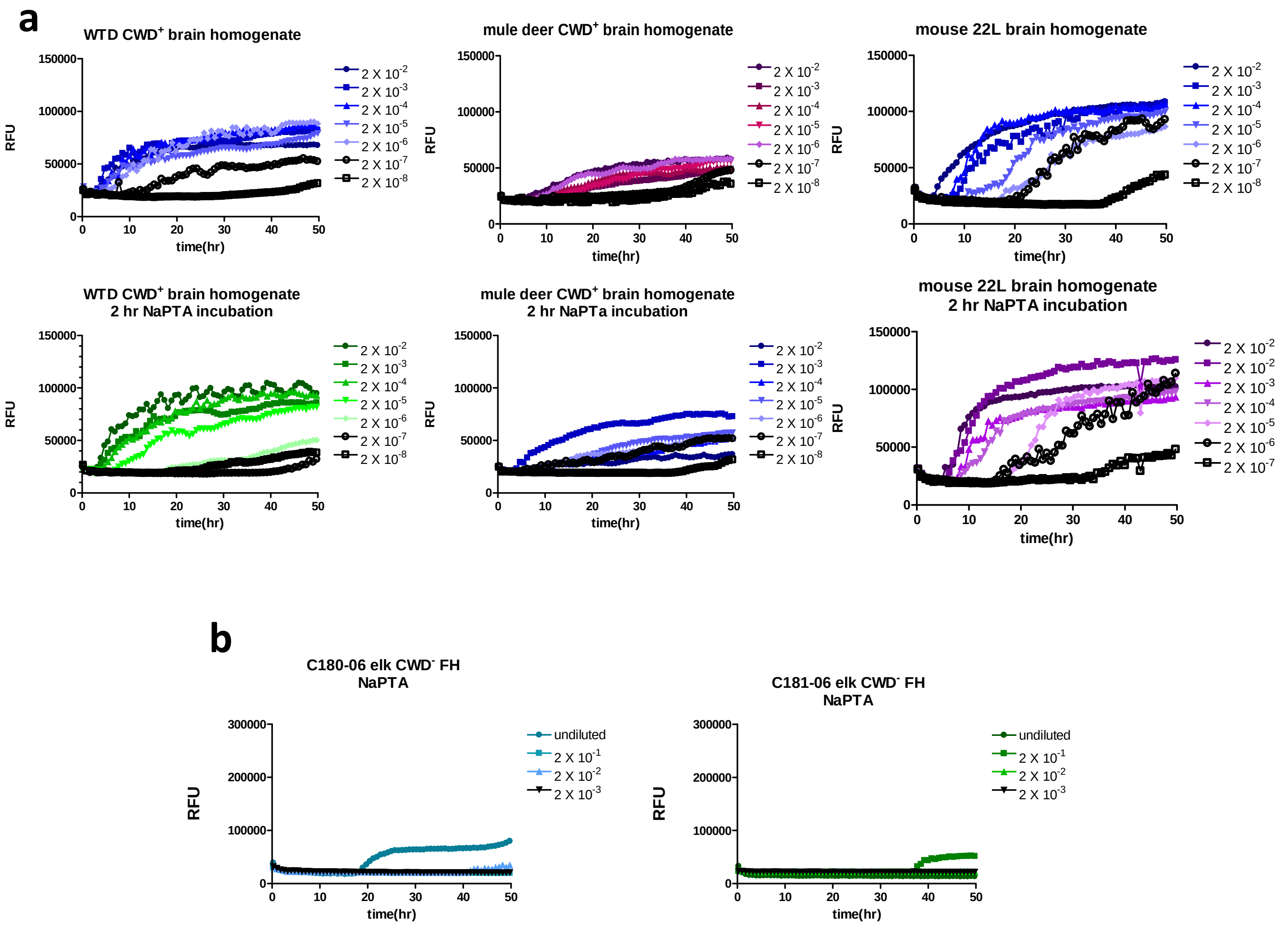

NaPTA precipitation does not inhibit RT-QuIC detection of prion seeds in infected brain homogenates. (a) Prion-infected brain homogenates of white-tailed deer, mule deer and mouse were subjected to NaPTA precipitation (lower panel) or not (upper panel). NaPTA precipitates were re-suspended in the original volumes. NaPTA-precipitated samples and non-precipitated brain homogenates were serially diluted, and aliquots of dilutions were used to seed RT-QuIC reactions in quadruplicate. Deer rPrP was used as a substrate. **(b)** Ten % fecal homogenates of CWD-negative elk were subjected to NaPTA precipitation and 10fold concentration. Serial dilutions were tested by RT-QuIC with deer rPrP as a substrate. Reactions were set up in quadruplicate, average ThT fluorescence is shown over the time course of the reaction.
