# Supplementary material for: Early and Non-Invasive Detection of Chronic Wasting Disease Prions in Elk Feces by Real-Time Quaking Induced Conversion
Source: PLoS One. 2016 Nov 9;11(11):e0166187. doi: 10.1371/journal.pone.0166187 (PMC5102397; doi:10.1371/journal.pone.0166187)
Supplement: S3 Fig — As further negative controls, fecal homogenates of CWD-negative deer were subjected to NaPTA precipitation and 10fold concentration. Serial dilutions were used to seed RT-QuIC reactions with mouse rPrP and substrate replacement. Reactions were set up in quadruplicate, average ThT fluorescence is shown over the time course of the reaction. (PDF) [file pone.0166187.s003.pdf]

S3 Figure

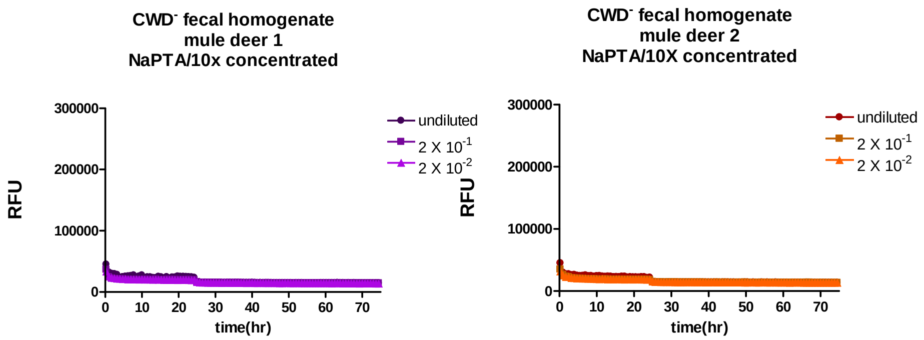

RT-QuIC with fecal homogenates of CWD-negative deer. As further negative controls, fecal homogenates of CWD-negative deer were subjected to NaPTA precipitation and 10fold concentration. Serial dilutions were used to seed RT-QuIC reactions with mouse rPrP and substrate replacement. Reactions were set up in quadruplicate, average ThT fluorescence is shown over the time course of the reaction.
